# Synergistic and redundant information dynamics are modulated by Alzheimer’s disease and cognitive impairment

**DOI:** 10.64898/2026.02.18.706630

**Published:** 2026-02-19

**Authors:** Keenan J. A. Down, Jonathan Huntley, Pedro A. M. Mediano, Daniel Bor

**Affiliations:** 1 Department of Psychology, Queen Mary University of London; 2 Department of Psychology, University of Cambridge; 3 Department of Computing, Imperial College London; 4 Department of Health and Community Sciences, University of Exeter; 5 Division of Psychology and Language Sciences, University College London

**Keywords:** Alzheimer’s disease (AD), partial information decomposition, integrated information decomposition

## Abstract

**Background:**

The early diagnosis of Alzheimer’s disease (AD), a cause of progressive cognitive decline, remains challenging. Recent information-theoretic advances allow brain dynamics to be quantified in terms of how regions share and combine information. Integrated Information Decomposition (ΦID) separates redundant (the same content present in multiple regions) from synergistic information (new content that emerges only when regions are considered together). Such information-dynamic measures may provide biomarkers relevant to AD risk and progression.

**Methods:**

Here we applied integrated information decomposition (ΦID) to resting-state fMRI from the Alzheimer’s Disease Neuroimaging Initiative (ADNI), to test whether ΦID measures are diagnostically sensitive and track cognition along the AD spectrum. For each region, we computed total synergy and redundancy and compared values across cognitively normal (CN), mild cognitive impairment (MCI), and AD groups.

**Results:**

Compared to CN, AD patients showed a striking synergy reduction across the entire brain, in concert with widespread redundancy increases, particularly in the executive and default mode networks. Transitions from CN to AD included an intermediate MCI decrease in redundancy, possibly reflecting early disease compensation strategies. This AD informational shift from complex higher level information processing to more robust inefficient forms likely reflects a cognitive shift to simpler, less integrative cognitive processes. Indeed, when re-analysing the data according to a standard cognitive clinical test (the Montreal Cognitive Assessment), we found a synergy-redundancy shift in high versus low performers broadly very similar to the CN to AD shift.

**Conclusion:**

AD shows a clear information-processing signature: reduced global synergy and increased redundancy, especially in the executive control network. These striking results provide powerful insights into the widespread information processing reconfiguration that occurs in AD, with clear changes already emerging at the earlier MCI stage. Further, these results provide a novel route to support early diagnosis and stratification.

## 1 Introduction

Alzheimer’s disease (AD) is the leading cause of dementia and is associated with progressive cognitive decline and impairment in the activities of everyday life [48, 69]. AD imposes substantial personal and societal costs [9, 41], however it can be challenging to accurately diagnose at early or preclinical stages [3]. Biomarkers are increasingly used to support early diagnosis [30, 31], and new biomarkers should combine early detection with a mechanistic explanation for cognitive decline.

Information theory has the potential to serve these dual goals [60]. Beyond connectivity strength or linear coupling, information theory can characterise how brain regions exchange and combine information [20, 21, 62]. One tool of contemporary information theory, Integrated Information Decomposition (ΦID) [44], formally separates interactions into redundant (the same information present in multiple regions) and synergistic (information that emerges only when regions are considered together, i.e., not recoverable from parts alone) components. The information-theoretic approach has been applied to understanding many cognitive effects, both in healthy and in clinical populations, including some work exploring AD [8, 22, 39, 43, 45, 46, 53].

In the current work, we directly test the potential for ΦID [44], a method for exploring information dynamics, to provide mechanistic insight and diagnostic utility, using resting state fMRI data from the Alzheimer’s Disease Neuroimaging Initative (ADNI). ΦID has been applied to resting state data from the Human Connectome Project (HCP), where it was shown to be strongly related to both structure and function in healthy individuals [39, 64], and we apply here it to individuals with AD, those with mild cognitive impairment (MCI), and a cognitively normal (CN) group.

## 2 Methods

### Alzheimer’s Disease Neuroimaging Initiative

Data used in the preparation of this article were obtained from the Alzheimer’s Disease Neuroimaging Initiative (ADNI) database (adni.loni.usc.edu). The ADNI was launched in 2003 as a public-private partnership, led by Principal Investigator Michael W. Weiner, MD. The primary goal of ADNI has been to test whether serial magnetic resonance imaging (MRI), positron emission tomography (PET), other biological markers, and clinical and neuropsychological assessment can be combined to measure the progression of MCI and early AD.

Participants with at least one available 10-minute resting state fMRI scan prior to November 22nd 2023 were selected from the ADNI database, giving a total number of 2907 scans. The ADNI data collection has consisted of multiple phases, ADNI 1, ADNI GO, ADNI 2, ADNI 3 and ADNI 4. Of these acquisition rounds, resting state fMRI acquisition became standard from ADNI 3 onwards (2017) and remains part of the ADNI 4 protocol (2022).

One of the limitations of the ADNI data is that many participants entered the study in different phases, so fMRI scans are unavailable at baseline for a large portion of the dataset. For the purpose of our analysis, we opted to instead select the most recent scan acquisition available for each participant. Although many participants had several scans, potentially allowing for a longitudinal analysis, we opted in this case only to select one scan per participant, due to the aforementioned variation in scan procurement. A breakdown between the three diagnostic groups CN, MCI, and dementia, presumed AD, is given in the table below. We note, in particular, that a sizeable number of scans selected did not have diagnostic data available at the point of the scan and were excluded.

**Table:** 

	No diagnostic data	CN	MCI	AD	Total
All scans	179	1322	1053	353	2907
Participants (scans selected)	0	568	369	181	1118

### ADNI functional data acquisition

From ADNI2 onwards, a 10-minute resting state functional MRI acquisition was performed at every in-clinic visit. For ADNI2 this was solely on Philips scanners. For ADNI3 and ADNI4, all scanning was done on 3T MRI machines with two different protocols; basic (TR = 3 seconds for machines without multiband imaging) and advanced (TR = 0.6 seconds for machines with multi-echo capabilities). Participants were scanned with eyes open. For more information and individual scanner configurations see https://adni.loni.usc.edu/methods/documents/. For greater statistical power we used both the basic and advanced multiband images in our analysis, down-sampling the advanced protocol so that all sampling was done at an equivalent TR of 3 seconds.

### Conversion to BIDS format

We used Clinica’s ADNI-to-BIDS tool [56, 57] to convert the ADNI dataset to the Brain Imaging Data Structure [24], a standardised data structure for storing neuroimaging data and its derivatives. The BIDS structure was designed with the goal of increasing reproducibility within neuroimaging studies.

The raw and converted data were stored and processed on Queen Mary University of London’s High-Performance Computing cluster, Apocrita, supported by QMUL Research-IT http://doi.org/10.5281/zenodo.438045.

### Preprocessing with fMRIPrep

We used fMRIPrep 23.0.2 [16, 17], a tool based on Nipype [23, 25] to preprocess the anatomical and functional data for all groups. fMRIPrep provides a standardised pipeline for functional MRI processing using the BIDS data structure [24], and performs both anatomical and functional preprocessing steps. As a BIDS app, fMRIPrep is designed to make comparisons between studies more straightforward by standardising processing pipelines.

The anatomical preprocessing pipeline corrects field non-uniformity, strips the skull, segments and normalises onto MNI152Lin standard space with a resolution of 2mm. The functional pipeline estimates head motion (including three rotation and three translation confound parameters), before applying spatiotemporal filtering and slice-time correction, where possible. White matter, grey matter and CSF signals were extracted in addition to component-based noise features (tCompCor and aCompCor [4]) up to 50% of the variance. Finally, the functional data was resampled onto MNI152Lin standard space.

For full details on both the anatomical and functional preprocessing we refer to the boilerplate output from fMRIPrep given in the supplementary materials in appendix.

### Further denoising and cleaning steps

Following the work in healthy participants by Luppi et al. [39], we regressed out the first five anatomical CompCor [4] components for both cerebrospinal fluid and white matter, along with translations and rotations for all three dimensions and their derivatives as was made available through confound outputs from fMRIPrep. We also regressed out a non-steady state component provided by fMRIPrep. Also following the work by Luppi et al., we performed a band-pass filter between 0.05Hz and 0.0025Hz. No blurring step was performed so as to prevent “information leakage” between regions, which would falsely indicate shared information between nearby regions. As ADNI uses two main fMRI acquisition protocols (**basic** with a TR = 3 and **advanced** with TR = 0.6), we downsampled slices so that, to the nearest slice, all scans had a comparable TR of about 3s.

### Brain parcellations

We used the Schaefer-200 and Schaefer-100 parcellations [59] to parcellate the fMRI images into ROIs. These were supplemented with additional sub-cortical regions (32 and 16) respectively [61], to recreate the extended Schaefer atlases used on previous work with healthy participants [39], giving parcellations of size 232 and 116 respectively, which we refer to as ‘extended Schaefer’ atlases. These parcels were categorised according to the 7 networks of Yeo et al. [71], along with the subcortical regions as an eighth collection to give 8 networks/macroscopic regions, which we use repeatedly in our analyses.

### BOLD time series extraction

For each region of interest in the extended Schaefer-232 (116) atlas we used Matlab to select the corresponding voxels in each fMRI session and take an average at each time step across all voxels in the region, giving a new region-wise time series of BOLD signal.

### Partial Information Decomposition (PID)

Our analysis uses Integrated Information Decomposition (ΦID), a method built to extend an earlier method called Partial Information Decomposition (PID). The Partial Information Decomposition framework was proposed by Williams and Beer in 2010 [67] as a tool for understanding how information is stored in a system of variables. In particular, given a series of source variables $X_{1},\ldots ,X_{n}$, which we assume we have knowledge of, how much information do we receive about a target variable Y, and how do the source variables qualitatively represent it?

It might be that several of the source variables provide the *same* information about Y (called **redundant** information), or that they give different, **unique** information about the target. Interestingly, it is also possible that knowledge of multiple sources can provide more information than any subset of the sources alone. This information is called **synergistic** information, and it reflects a kind of *additional deductive capability*, over and above any source used singularly. In this work, we focus primarily on ΦID’s generalisation of the **redundant** and **synergistic** information.

Knowledge of all sources together provides the maximum amount of information possible about Y, and this is the **mutual information**

(1) $$I\left(X_{1},\ldots ,X_{n};Y\right)=H\left(X_{1},\ldots ,X_{n}\right)+H\left(Y\right)-H\left(X_{1},\ldots ,X_{n},Y\right),$$

which quantifies the total amount of information in bits that $X_{1},\ldots ,X_{n}$ provide about Y and vice versa [60]. As such, the contribution to knowledge of Y by all pieces of partial information (called **atoms**) should total the mutual information.

To compute these different atoms, a partial order is constructed on the information provided by sources. Information which is provided redundantly about Y by two collections of sources $A_{1},\ldots ,A_{j}$ and $B_{1},\ldots ,B_{k}$ is written

(2) $$Red\left(A_{1}\ldots A_{j},B_{1}\ldots B_{k};Y\right)=I_{\cap }\left(\left\{A_{1}\ldots A_{j}\right\}\left\{B_{1}\ldots B_{k}\right\};Y\right),$$

where the curly brackets signify information that is provided by all bracketed groups. The function $I_{\cap }$ is called a redundancy function or measure of intersection information: it quantifies how much information is shared by all bracketed groups about the target. If a subset $S\subset \left\{A_{1},\ldots ,A_{j}\right\}$ also shares information with $B_{1},\ldots ,B_{k}$, then a partial order can be given by

(3) $$\left\{S\right\}\left\{B_{1}\ldots B_{k}\right\}⪯\left\{A_{1}\ldots A_{j}\right\}\left\{B_{1}\ldots B_{k}\right\},$$

where the order ⪯ represents ‘informativeness’. That is, this shared information is inherited (in some sense) by a larger collection of variables. At each level, we expect that *more* information is made available about the target Y by adding more variables.

For this reason, the **change** as we go up the order ⪯ should reflect information which is exclusively provided at that level. For example, in a system of two source variables $X_{1}$ and $X_{2}$ and a target Y, the information uniquely provided by $X_{1}$ is given by

(4) $$Unq\left(X_{1};Y\right)=I_{\cap }\left(\left\{X_{1}\right\};Y\right)-I_{\cap }\left(\left\{X_{1}\right\}\left\{X_{2}\right\};Y\right)=I_{\partial }\left(\left\{X_{1}\right\};Y\right).$$

That is, information uniquely provided by $X_{1}$ about Y is all the information provided by $X_{1}$ about Y minus the information which is also given by $X_{2}$ about Y. The notation ∂ is used to signify the direct contribution of information at this level in the order which is not available below it.

This process, isolating the individual atoms by subtracting those contributions lower in the order, is called **Möbius inversion** [13], and allows us to decompose information into non-intersecting pieces. It is the primary method used in information decomposition and set-theoretic perspectives on information theory [15, 44, 67].

At the top of the order is the **synergistic** atom, which reflects to what extent a collection of variables provides more information than the sum of its parts. For two variables this is given by

$$\begin{array}{l} Syn\left(X_{1},X_{2};Y\right)\;=I_{\partial }\left(\left\{X_{1}X_{2}\right\};Y\right)\\ \;=I_{\cap }\left(\left\{X_{1}X_{2}\right\};Y\right)-I_{\partial }\left(\left\{X_{1}\right\};Y\right)-I_{\partial }\left(\left\{X_{2}\right\};Y\right)-I_{\partial }\left(\left\{X_{1}\right\}\left\{X_{2}\right\};Y\right). \end{array}$$

In our scenario, we decompose the joint evolution of a pair of regions $R_{t}^{1}$ and $R_{t}^{2}$ and their state after one time step (TR), $R_{t+1}^{1}$ and $R_{t+1}^{2}$ (recall that since the TR is 3 seconds, the variable observations are 3 seconds apart). That is, we decompose the **time-delayed mutual information**

(5) $$TDMI\left(R^{1},R^{2}\right)=I\left(R_{t}^{1},R_{t}^{2};R_{t+1}^{1},R_{t+1}^{2}\right).$$

Although the structure of the order is provided by the partial information decomposition framework, the exact choice of how to calculate redundancy $\left\{X_{1}\right\}\left\{X_{2}\right\}$ is hotly contested. Despite this, many partial information decomposition methods show similar behaviour for Gaussian systems— a PID referred to as minimum mutual information (MMI) [2, 39]. This PID sets redundant information as the lowest of the marginal contributions $I\left(X_{1};Y\right)$ and $I\left(X_{2};Y\right)$. Following Luppi et al., we also use this PID measure as brain activity is suitably Gaussian [5], following previous work in neural information processing. In the supplementary, we also revalidate our results using a ΦID approach due to Ince based on common change in surprisal [29].

For two source variables and one target variable, partial information gives rise to 4 new information quantities over and above classical information theory: the redundant information (shared between the sources about the target), two unique components (unique to each variable), and a synergistic component (available only by observing both source variables at the same time). These four pieces are further refined in integrated information decomposition (ΦID) before being used in our analyses.

We note briefly that other decompositions of information also exist with potential to characterise synergy and redundancy [14, 15, 55], along with some non-decomposition methods such as the *O*-information [54]. For this work, we used the ΦID method, an extension of PID, to describe synergy and redundancy, although other approaches are viable.

### Integrated Information Decomposition (ΦID)

The partial information decomposition approach in its current form only allows for a single target, and does not consider how information might be stored across multiple target variables. For this reason, Mediano et al. [44] introduced **Integrated Information Decomposition** (ΦID), to explore how information provided by the sources is stored by multiple target variables, hence broadening the *taxonomy of information dynamics*.

Integrated information decomposition can be thought of as describing a *forwards* and *backwards* partial information decomposition at the same time. To see how this works, consider two regions of interest $R^{1}$ and $R^{2}$. We have the forward PID redundancy (information in the present which is stored redundantly about the future),

$$Red\left(R_{t}^{1},R_{t}^{2};R_{t+1}^{1},R_{t+1}^{2}\right),$$

and we have the reverse PID redundancy (information in the present which is stored redundantly about the past),

$$Red\left(R_{t+1}^{1},R_{t+1}^{2};R_{t}^{1},R_{t}^{2}\right).$$

ΦID posits that we can reasonably ask how much information stored redundantly at time step t was also then stored redundantly at time step t+1, giving a double-redundancy atom $I_{\partial }\left(\left\{R_{t}^{1}\right\}\left\{R_{t}^{2}\right\}\to \left\{R_{t+1}^{1}\right\}\left\{R_{t+1}^{2}\right\}\right)$. For each combination of PID atom in the present and PID atom in the future, there is a corresponding ΦID atom, meaning that 16 such combinations are possible for a system of two targets and two sources. All together, providing a PID redundancy function and the ΦID double redundancy function is enough to specify all 16 atoms in the decomposition.

In this work, unless otherwise qualified, we refer to $I_{\partial }\left(\left\{R_{t}^{1}\right\}\left\{R_{t}^{2}\right\}\to \left\{R_{t+1}^{1}\right\}\left\{R_{t+1}^{2}\right\}\right)$ as **redundancy** and $I_{\partial }\left(\left\{R_{t}^{1}R_{t}^{2}\right\}\to \left\{R_{t+1}^{1}R_{t+1}^{2}\right\}\right)$ as **synergy** without further qualification. For later analyses based on the taxonomic decomposition introduced in the original ΦID paper, we use r to represent redundancy, x and y to represent information unique to the first and second region respectively, and s to represent synergistic information. We will occasionally make use of the notation r→r or rtr to designate information that was redundant in the past and in the future. For example, we write rts to represent information stored redundantly in the past which is stored synergistically in the future. We mostly focus on the redundancy and synergy atoms in this work as per Luppi et al.’s approach, and which can be used to characterise an information-functional hierarchy [39].

While ΦID-MMI guarantees the positivity of the redundancy and synergy atoms, many of the other atoms can occasionally take negative values. Some authors interpret this as an embedded misinformation behaviour, or representing a sort of natural mathematical dual to redundancy (shared ignorance) [18]. In analyses where these atoms appear we examine relative changes in these atoms between diagnostic or cognitive groups, rather than their absolute values. In other results we focus on the non-negative synergy and redundancy terms.

### Matrix of redundant and synergistic interactions

Following previous work by Luppi et al. [39] on data from the Human Connectome Project (HCP), all of the sixteen ΦID atoms were computed for every possible pair of ROIs for every participant for the extended 232 Schaefer atlas (also replicated for the extended 116 parcellation). Following Luppi et al., we decomposed the TDMI with ΦID between every pair of regions $R_{t}^{1},\;R_{t}^{2}$ and their state in the next echo $R_{t+1}^{1},\;R_{t+1}^{2}$, giving a matrix of information dynamics for each scan. These matrices are provided for the sts and rtr atoms in the appendix in section 6.7.

For results on diagnostic state we averaged these matrices across all participants in each diagnostic group — cognitively healthy participants, participants with MCI, and those with dementia at the time of the scan, giving three 232 × 232 (116 × 116) matrices depicting *average* values between each pair of ROIs for each atom (rtr, rtx, rty, rts, xtr, xtx, xty, xts, ytr, ytx, yty, yts, str, stx, sty, sts).

This gives a 232 × 232 matrix of information dynamics between pairs of regions, representative of the average information dynamics across each diagnostic group for a given atom. We then cleaned up these matrices by regressing out age, years in education, gender, and intracranial volume (available either from the ADNI dataset, or where ICV was not available, we imputed it using data generated by fMRIPrep).

The precise behaviour of the null distribution for ΦID atoms is difficult to determine in this case; although permutation testing would be optimal (e.g. in a manner similar to the approach taken in [37]), due to the large quantity of data, this was mostly intractable for our calculations. For that reason, we tested all pairwise changes between the CN and AD groups in the redundancy and synergy atoms using independent two-tailed *t*-tests at the α=0.05 level, accounting for multiple comparisons (232 × 232 × 16 = 861,184) using the Benjamini-Hochberg procedure for constraining the false discovery rate. This result is reported in the supplementary materials in section 6.7. Significant increases and decreases were separated from each other prior to plotting. Regions were also re-ordered so that regions in each of the 7 Yeo networks (and subcortical regions) are grouped together [71].

### Reduction to a regional measure

Following Luppi et al. [39], for each pair of regions of interest (ROIs) in the Schaefer parcellations, we computed all of the ΦID atoms and obtained an average information dynamics matrix for each participant group as above. In results 3.1, 6.3, and 6.10, we took the sum across each column of this matrix to reduce these pairwise interactions to interpretable behaviour at a regional level. For example, to compute the rtr for a given region $R^{i}$, we summed down the matrix column:

(6) $$Redundancy\left(R^{i}\right)=\sum_{j}\;I_{\partial }\left(\left\{R_{t}^{i}\right\}\{R_{t}^{j}\},\left\{R_{t+1}^{i}\right\}\{R_{t+1}^{j}\}\right).$$

(7) $$Synergy\left(R^{i}\right)=\sum_{j}\;I_{\partial }(\left\{R_{t}^{i}R_{t}^{j}\},\{R_{t+1}^{i}R_{t+1}^{j}\right\}).$$

All self-measures ($R^{i},R^{i}$) were taken to be zero. Comparisons were done regionwise using an FDR-corrected Welch’s t-test.

### Intranetwork group comparisons

In addition to comparing the total summed redundancy and synergy for each region with all other regions, we also performed a network-wise analysis by averaging all intranetwork redundancy and synergy within each network. For regions i in some set of regions corresponding to a network S, we computed

(8) $$Red(S)=\frac{1}{|S{|}^{2}}\sum_{i,j\in S}\;Red\left(R^{i},R^{j}\right)$$

(9) $$Syn(S)=\frac{1}{|S{|}^{2}}\sum_{i,j\in S}\;Syn\left(R^{i},R^{j}\right),$$

before using FDR-corrected Welch’s *t*-tests to detect statistically significant ΔRed and ΔSyn at the α=0.05 significance level.

### Regional *z*-scoring against the CN group

For the qualitative depiction in result 3.1, we selected the average summed synergy and redundancy atoms from each region across the MCI and AD groups (repeating in the supplementary with the Medium MoCA and Low MoCA), before *z*-scoring them against that region’s summed measures across all scans in the CN group (or the High MoCA group, respectively). That is to say, each region’s redundant and synergistic activity in MCI/AD (Medium/Low MoCA) is scored based on how unusual its value would be in the CN distribution. These *z*-scores were then plotted against each other in result 3.1 to show a qualitative picture of changes in information dynamics.

Note this is done region-wise. For each MCI/AD region, the CN mean for the summed atom of that region is subtracted to give a mean of zero, before being divided by the deviation for that region to normalise onto a *z*-score.

### Projection onto the first principal component

In result 3.1 we also project synergy and redundancy onto the first axis of variation. This was done in python by projecting the variation in synergy and redundancy onto its first component using NumPy. Regions were coloured on this plot based on their location in the 7 Yeo networks (or if that region was a supplementary subcortical region). The variance explained in each diagnostic (cognitive) group by PC1 was also extracted.

### Cortical plotting

**Figure 1.**
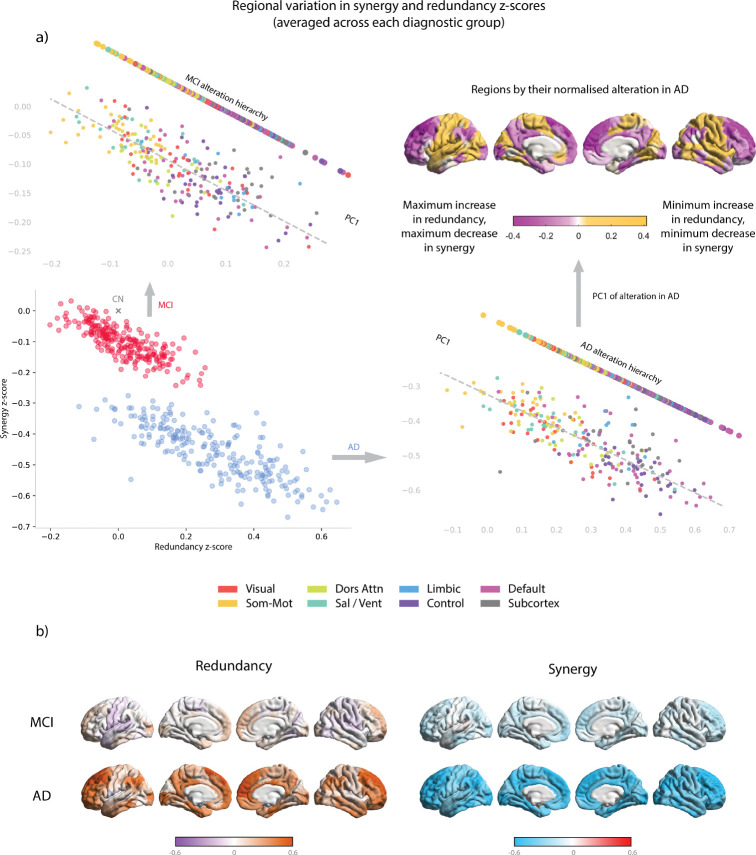
Information dynamics are altered according to functional roles. Figure a) Bottom left: 232 regions from the extended Schaefer parcellation averaged across all participants labelled as having MCI (red) and dementia (blue). Regions are z-scored against the CN mean (grey cross) for each region in their total amount of redundancy and synergy. Top left: z-scores for regions from the MCI group, with Yeo networks labelled (subcortical regions depicted in grey). This is then projected onto the first principal component of variation across all 232 regions to show that changes in information dynamics are modulated by the unimodal-transmodal axis. Bottom right: as with the top left, but with the AD group. Top right: each region is plotted onto the cortex with its first principal component (PC1) scores from the AD *z*-scores. Regions with maximum increases in redundancy and maximum decreases in synergy are in pink, minimum increases in redundancy and minimum decreases in synergy are in yellow. Figure b) Redundancy and synergy *z*-scores given separately. Left: regions with increasing redundancy are given in orange; regions with decreasing redundancy are given in pink, and increases in orange. Right: regions decreasing in synergy are highlighted in blue; increases are depicted in red.

Cortical plots were created using the ENIGMA toolbox’s plot_cortical function in Matlab with custom colour palettes [36]. In figures 1 a) and 4 a), the cortical plot is the first principal component of *z*-scored changes to redundancy and synergy. In figure 1 b) and 4 b), the *z*-scores are used in redundancy and synergy, respectively separated.

**Figure 2.**
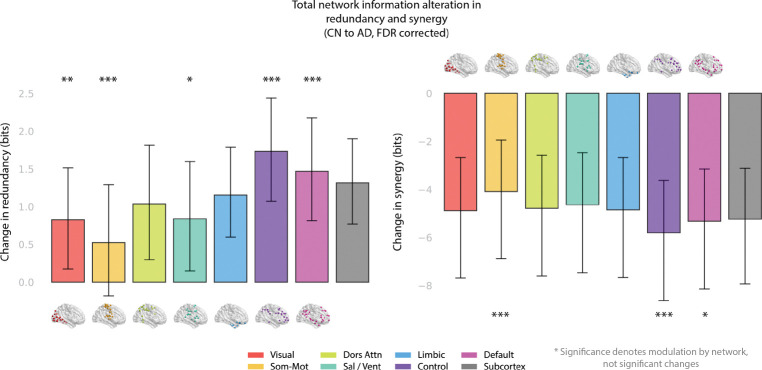
Information changes from CN to AD are heterogeneous across brain networks. For each region the total redundancy and synergy with all other brain regions is calculated, before being averaged across each network to find the average total increase or decrease in that network’s average regional information processing. Left: increases in total redundancy averaged across each of the Yeo networks (plus subcortex); right: decreases in total synergy averaged across each of the Yeo networks. Significant deviations from a global average were computed using a permutation test with *n* = 10,000 resamples and FDR correction to account for multiple comparisons, shuffling network labels rather than diagnostic labels to test the hypothesis that changes in information dynamics (redundancy or synergy) are modulated by region, not solely by a global effect. Significances are illustrated at the $\alpha =0.05^{*},0.01^{**}$, and $0.001^{***}$ levels respectively. Confidence intervals were computed by bootstrap resampling to obtain the 95% confidence interval.

For result 3.1 and figures 2 and 9 we used plots from [43], originally constructed with Brainnetviewer [70], with some recolouring.

### Verifying functional modulation of information processing changes

In result 3.1 and figures 2 and 9 we averaged the summed measure across each of the 8 macroscopic network regions (7 Yeo networks, plus 32 subcortical ROIs). To test whether function (and not a whole-brain effect) modulated the synergistic decrease and redundant increase when comparing CN to AD, we used a permutation test with *n* = 10,000 resamples, shuffling network labels. An FDR correction was also applied for the 8 tests performed for synergy and redundancy respectively.

### Taxonomic information signature calculation

The taxonomic calculation in result 3.3 used the intranetwork values for individual ΦID atoms, grouped together in a fashion similar to the modes (taxa) described in the paper introducing ΦID [44]. Depending on the qualitative behaviour of the individual atoms, they can be grouped into 6 broad modes:

**Table:** 

Mode	Atoms	Interpretation

Storage	rtr, xtx, yty, sts	Structure completely preserved over time
Copy	xtr, ytr	Data is copied into two regions over time
Transfer	xty, ytx	Data exchanged by two regions
Erasure	rtx, rty	Duplicated data loses its duplicate
Downward causation	stx, sty, str	Higher-order features become lower-order
Upward causation	xts, yts, rts	Lower-order features becomes higher-order

In principle these quantities can all vary independently of each other, but due to additional constraints imposed by the MMI-ΦID, the minimum unique information in either direction must be precisely zero by construction. This gives

(10) $$\left\{\begin{array}{l} xtr+xts+xtx+xty=0\;or\\ ytr+yts+ytx+yty=0 \end{array}\right.$$

and

(12) $$\left\{\begin{array}{l} rtx+stx+xtx+ytx=0\;or\\ rty+sty+xty+yty=0. \end{array}\right.$$

Overall, for any pair of signals $R^{1}$ and $R^{2}$, 8 of the 16 ΦID atoms calculated between those two signals with MMI-PID will, assuming non-negativity, be zero. In practice this is frustrated by negativity considerations, but the total number of degrees of freedom is certainly reduced.

In empirical practice we found that the copy and transfer atoms (dual to each other in direction) and the upwards and downwards causation modes (also dual) behaved structurally very similarly. For this reason, we grouped into four dominant modes, **storage**, **transfer**, **copy/erasure**, and **multi-scale causation**.

By summing up the corresponding atoms, we computed intranetwork values for each mode in each diagnostic (MoCA) group. For each mode-network pair, we tested for modulation across groups using a one-way ANOVA with an FDR correction across all 32 tests, reporting at the α=0.05,0.01,0.001 levels. The same test was applied to MoCA categories, with results given in the supplementary.

Post-hoc tests were performed on pairs of groups (pairs of diagnostic groups or MoCA categories) for each one-way ANOVA which was significant, using Welch’s *t*-test to compare means without assumptions of variances, adjusted for the false discovery rate at the α=0.05 level. Significant differences are reported in tables 5 and 6.

## 3 Results

### 3.1 A strong principal axis captures heterogenous synergy and redundancy changes to ROIs in AD

#### 3.1.1 Organisation of changes in summed information dynamics across diagnoses

In order to qualitatively depict how redundancy and synergy are changed in different ROIs and networks when comparing CN, MCI, and AD groups, we computed the mean synergy and redundancy, along with their standard deviations, for each ROI across the CN group. Using these values, we *z*-scored the average synergy and redundancy for each ROI from the MCI and AD groups, giving an indication of how each region changed relative to a cognitively healthy baseline. A corresponding plot of these scores can be seen in figure 1, where each dot represents an ROI from either the average MCI information dynamics or average AD information dynamics. Network-wise *z*-scores are available in the supplementary materials.

In figure 1 a) we show the regional change in information processing dynamics between the CN group and the MCI and AD groups. For each region i, the synergistic information and redundant information are summed across all interactions with all other regions j, giving a single measure of a region’s synergistic and redundant behaviour. For scans where the individual is labelled as CN, the mean and standard deviation was computed for each region’s redundancy and synergy totals. The average regional synergy and redundancy in all MCI (red) and AD (blue) acquisitions was found and *z*-scored against the CN mean and standard deviation for that region. The corresponding scores are plotted against each other in the bottom left. In order to better see the function of each region, the top left and bottom right panels show the same regions, now coloured with each of the Yeo networks [71] (plus an additional subcortical grouping [61]), along with a projection onto the first principle component (PC) of variation. In the top right of 1 a) there is a cortical plot of the AD group projected onto the first principal component (PC1), showing that greatest regional change is observed in the limbic, ECN, and DMN systems. Figure 1 b) shows the synergy and redundancy *z*-scores independently of each other, plotted onto the cortex.

When arranging brain regions in this way (changes in redundancy and synergy), variation occurs mostly across a single axis, with the first principal component capturing 92.3% of the regional variation in MCI and 93.2% of variation in AD.

We repeated this procedure with three categories based on the Montreal Cognitive Assessment (MoCA) [47], with results pointing to a similar structure. The corresponding results are available in the supplementary materials in section 6.1.2.

#### 3.1.2 Changes between CN and AD

When looking at individual ROIs, we applied Welch’s *t*-test to each of the 232 parcels and applied an FDR correction to test for significant differences between the CN and AD groups. At the α=0.05 level, all 232 regions from all networks exhibited a significant decrease in synergy. In redundancy, a majority of executive control network (ECN) regions (29/30), default mode network (DMN) regions (39/46), subcortical regions (27/32), and limbic regions (10/12) exhibited a significant increase in the AD group compared to the CN group. Significant increases in the same comparison were also observed in the dorsal attention network (11/26 ROIs), visual system (10/29 ROIs), salience network (6/22 ROIs), and somatomotor network (3/35 ROIs), but with lower coverage of the network seeing a significant increase.

In addition to the individual ROIs, we also performed analyses on the intranetwork average (looking at pairwise interactions inside of each network). We observed a significant decrease in the AD group compared to the CN group in redundant interactions in the somatomotor network (ΔRed=-0.009 bits, FDR-adjusted q=0.004, Hedges’ g=-0.242), and in synergistic interactions in the visual (ΔSyn=-0.018 bits, q=0.008,g=-0.290), dorsal attention (ΔSyn=-0.016 bits, q=0.013,g=-0.267), salience (ΔSyn=-0.012 bits, q=0.046,g=-0.215), limbic (ΔSyn=-0.019 bits, q=0.003,g=-0.349), control (ΔSyn=-0.026 bits, q<0.001, g=-0.498), and default mode networks (ΔSyn=-0.020 bits, q=0.002,g=-0.387), along with the subcortex (ΔSyn=-0.023 bits, q<0.001, g=-0.446). Statistically significant increases in the AD group compared to the CN group were found in redundancy in the limbic (ΔRed=0.005 bits, q=0.036,g=0.214), control (ΔRed=0.008 bits, q<0.001, g=0.416), and default mode networks (ΔRed=0.004 bits, q=0.030,g=0.223), as well as in the subcortex (ΔRed=0.005 bits, q=0.008,g=0.280).

#### 3.1.3 Changes between CN and MCI

Before applying an FDR correction, (5/32) regions in the subcortex, (6/32) in the default mode network, (3/39) in the executive control network, (1/12) in the limbic network, and (1/29) in the visual network exhibited significant increases in redundancy in MCI when compared to the CN. Similarly, (8/12) regions in the limbic system, (15/32) in the subcortex, (14/30) in the ECN, (20/46) in the DMN, (5/29) in the visual network, (4/26) in the dorsal attention network, and (1/22) regions in the salience network showed decreased synergy in MCI when compared to the CN group, but none of these results (redundancy or synergy) survived the FDR correction.

When performing an analysis on intranetwork averages inside of each of the 8 networks, we observed only one significant difference surviving FDR correction, which was a decrease in redundancy in the somatomotor network in the MCI group when compared to CN individuals (ΔRed=-0.009 bits, q<0.001, g=-0.269). Prior to FDR correction, a decrease was also detected in redundancy in the salience network along with an increase in synergy in the somatomotor network, but these did not survive the multiple-comparisons correction.

#### 3.1.4 Changes between MCI and AD

When comparing the MCI to the AD group with an FDR correction, we saw an increase in redundancy in AD in (23/30) regions in the ECN, (35/46) in the DMN, (13/26) in the dorsal attention network, (15/32) in the subcortex, (9/22) in the salience network, (8/35) in the somatomotor network, (6/29) in the visual network, and (2/12) in the limbic system. Similarly, we saw significant decreases in synergy in AD in all regions (30/30) in the ECN, (34/35) in the somatomotor network, (25/26) in the dorsal attention network, (21/22) in the salience network, (29/32) in the subcortex, (40/46) in the default mode network, (25/29) in the visual system, and (9/12) in the limbic system. No increases in synergy or decreases in redundancy survived FDR correction.

For the intranetwork average comparison, we found significant increases in redundancy in AD compared to MCI in the executive control network (ΔRed=0.008 bits, q=0.005,g=0.344), default mode network (ΔRed=0.005 bits, p=0.021,g=0.269), and subcortex (ΔRed=0.004 bits, q=0.043,g=0.213), while we observed significant decreases in synergy in AD compared to MCI in the visual (ΔSyn=-0.016 bits, q=0.035,g=-0.251), dorsal attention (ΔSyn=-0.015 bits, q=0.035,g=-0.242), limbic (ΔSyn=-0.014 bits, q=0.035,g=-0.245), control (ΔSyn=-0.020 bits, q=0.005,g=-0.361), default mode networks (ΔSyn=-0.017 bits, q=0.016,g=-0.311), along with the subcortex (ΔSyn=-0.017 bits, q=0.014,g=-0.316).

### 3.2 Synergy loss and redundancy gain in AD are not uniform across networks

All *p*-values in this section are given with FDR correction. Taking averages across all of the 7 Yeo networks plus the subcortical collection of atoms, significant decreases in total synergy between the CN group and the AD group were observed in all 8 brain networks with effect sizes largest in the executive control network (ECN) (Welch’s t=-4.509, FDR-adjusted q<0.001, Hedges’ g=-0.510), subcortex (t=-4.215, p<0.001, g=-0.475) and default mode networks (DMN) (t=-4.135, q<0.001, g=-0.467). There were still moderate effect sizes in the limbic (t=-3.785, q<0.001, g=-0.433), visual (t=-3.731, q<0.001, g=-0.415), dorsal attention (t=-3.666, q<0.001, g=-0.402), and salience networks (t=-3.610, q<0.001, g=-0.397). The smallest change was seen in the somatomotor network, although this difference was still strongly significant (t=-3.220,q=0.001,g=-0.349).

When looking at total redundancy across each Yeo network, the ECN once again showed the strongest effect when comparing CN to AD (Welch’s t=4.948, FDR-adjusted q<0.001, Hedges’ g=0.491), followed by the subcortical (t=4.484,q<0.001,g=0.448), default mode (t=4.169,q<0.001,g=0.419), and limbic (t=3.707,q<0.001,g=0.408) networks. Unlike for synergy, there is then a strong drop off in effect size. Despite showing a smaller overall effect, changes were still significant in the dorsal attention (t=2.668,q=0.013,g=0.244), visual (t=2.408,p=0.022,g=0.225), and salience (t=2.261,q=0.028,g=0.209) networks. The changes to redundancy in AD compared to CN did not reach the threshold for significance in the somatomotor network (t=1.379,q=0.169,g=0.124).

Changes in synergy and redundancy for each network, along with a different statistical test for network-specificity, are shown in figure 2, with error bars found using bootstrap resampling for a 95% confidence interval.

#### 3.2.1 Network modulation is not simply due to a global effect

To explore the idea that increases in redundancy and decreases in synergy are inhomogeneous across brain **networks**, we tested the hypothesis that individual Yeo networks exhibited their own information dynamics in each group (rather than a global effect across all brain regions) using a permutation test, shuffling synergy and redundancy changes across ROIs. Results are shown for the 7 Yeo networks plus an additional collection of subcortical regions in figure 2.

When comparing the CN group to the AD group, the change observed is more homogeneous across brain regions. Network changes in synergy which could not be explained by a global effect appeared in the ECN (ΔSyn=-5.806, FDR-adjusted q<0.001), the DMN (ΔSyn=-5.324, FDR-adjusted q=0.009), and the somatomotor network (ΔSyn=-4.092, FDR-adjusted q<0.001).

Looking at the difference in redundancy, we saw a more heterogenous change across brain regions when comparing the CN group to those with AD. Once again the executive control network (ECN) saw the largest increase (ΔRed=1.733, FDR-adjusted q<0.001), followed by the default mode network (DMN) (ΔRed=1.469,q<0.001). Also significantly deviating from a global shift were the salience (ΔRed=0.839,q=0.032), visual (ΔRed=0.826,q=0.008), and somatomotor networks (ΔRed=0.525,q<0.001), which saw a smaller increase than the global network mean.

### 3.3 Changes in taxonomic information signatures in progression to AD and cognitive decline

One of the main strengths of ΦID is that atoms can be grouped together into larger *modes* which describe different classes of dynamic information processing. In order to better understand the mechanisms underlying the observed increase in redundancy and decrease in synergy, we used groups of atoms from the paper introducing ΦID to explore the information dynamics observed [44]. Due to the assumed stationarity of the distribution, we have simplified the original modes to four: *multi-scale causation*, *copy and erasure*, *transfer*, and *storage*.

The *multi-scale causation* mode reflects the amount of higher-order information representations and lower-order representations which are exchanged. In terms of cognition, we believe this to be directly related to the stability of different information representations in the brain. Where transitions into and out of higher-order representations are frequent, long chains of deductive information representations are less common.

The *copy and erasure* mode reflects where information is both increasingly duplicated and more often deleted^1^. In terms of cognition and brain dynamics, we expect this might correspond to a more macroscopic propagation of information and a delocalisation of specific information transmission; complexity in representations likely decreases.

The *transfer* mode describes information that was relocated from one region to another, without duplication. In terms of brain dynamics, this corresponds to a ‘clean information flow’ from region to region. Reduction in transfer likely represents difficulties with neuronal signal transmission, with signal often being dropped.

Finally, the *storage* mode represents information that remained in the same atom between the two time steps. In terms of neural representations, this corresponds to information that is persistently and robustly encoded, and not dropped or otherwise interfered with.

**Figure 3.**
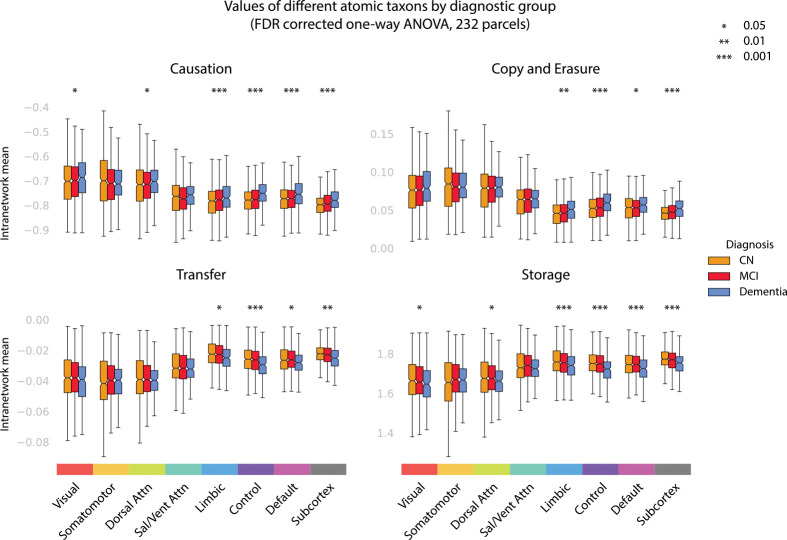
Taxonomic information processing is strongly modulated by diagnostic group in limbic, control, default mode and subcortical regions, with strong decreases in transfer and storage in AD. Four macroscopic modes (multi-scale causation, copy and erasure, transfer, and storage) were averaged across all pairs of brain regions inside of 7 Yeo networks using the ΦID approach, along with a collection of subcortical regions. The upwards and downwards causation modes are grouped together into a **multi-scale causation** mode, and the copy and erasure modes are also grouped due to linear constraints. For each mode-network pair, we performed a one-way ANOVA, correcting for the FDR.

For each of the 7 Yeo networks (along with the additional subcortical areas), atoms inside of each mode (multi-scale causation, copy and erasure, transfer, and storage) were added together and averaged across all of the ***intranetwork*** interactions for those regions. For example, the *transfer* mode in the *limbic system* was found by averaging all of the xty and ytx (transfer) atoms between all pairs of regions within the limbic system and adding them together. The results, given here with an FDR-corrected one-way ANOVA, are shown in figure 3.

Across diagnostic groups, the multi-scale causation and storage modes saw the most significant results, with the omnibus ANOVAs locating a statistically significant group variation at the p<0.001 level in the ECN, DMN, subcortex, and the limbic system, following a similar pattern as previously observed in sections 3.1 and 3.2. In addition to this, in the multi-scale causation and storage modes, the visual and dorsal attention networks varied across groups at the p<0.05 significance level. In none of the four modes did the ANOVAs detect significant variation across groups in the somatomotor or salience networks.

Post-hoc Welch’s *t*-tests with FDR correction revealed that all of the group differences were driven by comparisons with the AD group, with no comparisons between the CN and MCI groups reaching the threshold for statistical significance. In the multi-scale causation and copy-erasure modes, significant increases were detected when comparing the AD group to the CN group, while in the transfer and storage modes, significant decreases were detected when comparing the AD group to the CN group.

The largest effects appeared when comparing CN to AD in the executive control network, which reached the p<0.001 threshold for significance in all four modes (increasing with AD in multi-scale causation and copy-erasure, decreasing in transfer and storage). Full results for the post-hoc Welch’s *t*-tests are given in supplementary in table 5.

#### 3.3.1 Correspondence between information signatures in cognitive examination and diagnosis

For all of the results available here, similar patterns emerged when using three groups (high, medium, and low cognition) based on the Montreal Cognitive Assessment (MoCA) [47], and are presented in the supplementary materials 6. Most notably, broad global decreases in synergy were seen in the low MoCA group when compared to the high MoCA group, along with broad increases in redundancy, although the spread of synergistic change was greater than when comparing diagnostic categories.

As in the diagnostic analysis, significant decreases in total synergy were found to be highly present in the ECN, salience network, DMN, subcortex, dorsal attention network, and somatomotor network, with the fewest ROIs showing reduction of synergy in low MoCA in comparison to high MoCA in the limbic system. Similarly, increases in redundancy from high to low MoCA were seen heavily in the ECN, subcortex, and DMN, with some increases also seen in the dorsal attention network, salience network, limbic, and visual systems. Very few ROIs in the somatomotor network showed similar increases with cognitive decline.

Finally, as with the diagnostic categories, changes in redundancy and synergy between high and low MoCA cognitive scores were seen to highly depend on brain network and could not be explained solely by a global effect. All results on the MoCA categories are reported in full in the supplementary materials 6.

## 4 Discussion

In this work we applied recent advances in the theory of information decomposition to study how the brain represents information in AD and MCI using the large ADNI fMRI dataset, exploring two main information-theoretic effects: synergy, where different regions cooperatively integrate information, and redundancy, where regions represent duplicate information. The AD group exhibited major decreases in synergy (in all regions) and increases in redundancy (in a majority of regions) when compared to the CN group, with similar prodromal patterns already appearing in the MCI group (although this effect did not survive FDR correction). Moreover, we found that regional changes observed fell strongly onto a principal axis, ranging from highly-associative regions in the ECN and DMN (which saw the greatest decreases in synergy and greatest increases in redundancy) to less associative networks (such as the somatomotor network), which saw smaller effects. More than this, we showed that all our results are similarly explained by cognitive deficits using categories derived from MoCA scores [47], directly linking information dynamics to cognition. Overall, this work strongly demonstrates the efficacy of the information-dynamical approach to understanding cognitive deterioration in AD, and opens new avenues to the construction of information-theoretic biomarkers of cognitive decline.

Most of our results are best explained by connectonomic disruption, especially where it corresponds to AD-related atrophy (for example, in a Braak-like staging pattern [6, 7]). Synergistic effects are often fragile [51], while redundant information, which exhibits greater informational stability, might survive the neuronal disruption associated with amyloid and tau deposition, or possibly be more heavily utilised in order to overcome information loss associated with neuronal dysfunction. Our finding that multi-scale causation and copy-erasure modes are increased in AD further supports this hypothesis, along with the destruction of the transfer and storage modes in cognitive decline, which further highlight the possibility of signal disruption.

These results have implications for how we link high-level cognition to lower-level qualitative information processing mechanisms, opening up new avenues for exploring early diagnosis of major neurocognitive disorders with imaging techniques. Synergy, in particular, signifies a kind of information-theoretic *deduction*, so the especially strong decrease in synergy in the executive control network is insightfully consistent: as it has been shown that higher synergy is associated with highly-associative brain regions employed in complex cognition in healthy individuals [39], the decrease in synergy observed here might correspond to a decreased ability to synthesise and integrate information from multiple sources. Such a decline could render complex cognition more challenging, and this effect might be visible even at the MCI stage with careful biomarker development. Since synergy is often represented as a measure of emergent behaviour, and is also seen to be reduced in loss of consciousness [38, 39, 40], this work also gives further credence to the classification of AD as a disorder of consciousness (DoC) [28].

In addition to the broad increased redundancy and decreased synergy effect, we also found that changes in information dynamics in MCI and AD (and across our MoCA categories) were modulated by Yeo network [61, 71], with somatomotor regions demonstrating the greatest resistance to changes in information dynamics in AD compared to CN. In the AD group, the greatest changes were seen in the executive control and default mode networks, with subcortical regions also strongly affected. This pattern roughly approximates a Braak-like staging pattern [6, 7]. In this pattern, early tau deposition is observed in limbic and subcortical regions before an isocortical presentation at later stages (though somatomotor regions remain least affected). This structural decline can also be seen in our results: in the MCI group, we note that, despite not surviving FDR correction, the limbic system, subcortex, and ECN showed the strongest changes when compared to the CN group. This connection supports the hypothesis that the cellular transport disruption associated with tau deposition is implicated in changes in information representation— an intriguing mechanistic insight warranting further investigation.

To explore the qualitative changes in information processing with a more mechanistic lens we also explored intranetwork information dynamics by grouping atoms into ΦID modes: multi-scale causation, copy-erasure, transfer, and storage. We saw increases in the multi-scale causation and copy-erasure modes and decreases in the transfer and storage modes when comparing the AD group with the CN group, with the strongest changes seen in the limbic system, ECN, DMN, and subcortex, with somatomotor and salience networks least affected. Decreases in the transfer and storage modes are strongly supportive of the idea that information transmission and persistence is damaged in AD, indicating a direct disruption of information flow and decreased signal persistency. In terms of behaviour, we saw that this could potentially correspond to many cognitive difficulties: if information flow around the brain is interrupted, it could be linked to forgetfulness, or difficulties following chain-of-thought reasoning. Cognitive representations that would normally be stable and persistent (such as when focussing or holding attention), could inadvertently be dropped if local information storage declines. More work using qualitative information theory could illuminate the relationship to specific cognitive deficits in dementia.

In addition to the decreased transfer and storage, the observed increase in multi-scale causation might be due to a reduced stability of representations; if information cannot be stored persistently in the same fashion, alternative representation mechanisms might need to be more frequently substituted in, adding to this volatility. Neural pathways might become noisier, potentially swapping in and out of deductive cognition to understand and represent mental calculations, objects, and experiences. Increases in copy-erasure dynamics might represent an increase in *transient* data representations, or, perhaps as a counteraction mechanism, an attempt to *flood* a signal through a disrupted connectome, even if this signal lacks persistence. Representations might be split across multiple regions, providing more widely experienced (across areas), but more easily forgotten, cognition, a potentially jarring combination. Exploring these dynamics and their relationship to macroscopic behaviour and cognition will prove fruitful for understanding qualitative differences in brain activity in those with AD and cognitive impairment, along with the first-person experiences of such diseases.

One limitation of the ADNI fMRI dataset is that acquisitions are resting state, with participants not actively engaged in any cognitive task. This means that the signature we have obtained is subject to a large amount of noise: at any time, participants might be wind-wandering, recalling memories, or otherwise transitioning between different mental states. Because of this, the results we report here are only significant at the group level and do not, without further analysis, offer individual diagnostic strength. We expect that future work on task-based paradigms in cognitive impairment might offer a more participant-specific signature of information processing (see [43], for example). Given our results, we expect that tasks targeting the ECN, DMN, subcortex, or limbic system might offer the most potential for the development of an information decomposition-based diagnostic tool.

An additional limitation in this work is ongoing mathematical speculation about partial and integrated information decomposition methods. Many versions of both decompositions exist (see, for example, Kolchinsky’s commentary [34]), and precise choices for how to define redundancy have not yet been fully evaluated in the literature. The current view is that redundancy, as a concept, can be defined in multiple ways, and we make no particular philosophical commitments in this work. Despite this, we did validate our analyses with two different ΦID approaches, with results available (showing consistent patterns to those found in the main body) in the supplementary materials in section 6.13.

Similar work has appeared in the literature due to Zhang et al. [72], however their analysis focusses primarily on the graph-theoretic properties of synergy and redundancy. Our analyses focus on atomic differences themselves. In particular, we explore how these measures change between CN individuals, those with MCI, and those with AD. In the supplementary materials we also explore three different categories of cognitive ability, using the Montreal Cognitive Assessment (MoCA).

Overall, this work shows that information decomposition methods hold great promise for the development of explainable biomarkers of cognitive decline, and we expect that relating information decomposition and specific cognition will be a highly insightful research trajectory, with especially promising insights for cognitive disorders and neuronal disease.

## Supplementary Material

Supplement 1

## Footnotes

1 For our analyses we have assumed distribution stationarity. As such, copy and erasure are linked; copy went up and not erasure, there would be a cascading imbalance in redundant representations, eventually corresponding to more information than the brain can physically represent. As such, erasure must also simultaneously increase. A similar effect links upwards and downwards causation.

## References

[1] Avants B., Epstein C., Grossman M., and Gee J.. Symmetric diffeomorphic image registration with cross-correlation: Evaluating automated labeling of elderly and neurodegenerative brain. Medical Image Analysis, 12(1):26–41, 2008. 17659998 10.1016/j.media.2007.06.004 PMC2276735

[2] Barrett A. B.. Exploration of synergistic and redundant information sharing in static and dynamical gaussian systems. Physical Review E, 91(5):052802, 2015. 10.1103/PhysRevE.91.052802 26066207

[3] Beach T. G., Monsell S. E., Phillips L. E., and Kukull W.. Accuracy of the clinical diagnosis of alzheimer disease at national institute on aging alzheimer disease centers, 2005–2010. Journal of neuropathology and experimental neurology, 71(4):266–273, 2012. 22437338 10.1097/NEN.0b013e31824b211b PMC3331862

[4] Behzadi Y., Restom K., Liau J., and Liu T. T.. A component based noise correction method (CompCor) for BOLD and perfusion based fmri. NeuroImage, 37(1):90–101, 2007. 17560126 10.1016/j.neuroimage.2007.04.042 PMC2214855

[5] Bím J., De Feo V., Chicharro D., Bieler M., Hanganu-Opatz I. L., Brovelli A., and Panzeri S.. A non-negative measure of feature-related information transfer between neural signals. BioRxiv, page 758128, 2019.

[6] Braak H., Alafuzoff I., Arzberger T., Kretzschmar H., and Del Tredici K.. Staging of alzheimer disease-associated neurofibrillary pathology using paraffin sections and immunocytochemistry. Acta neuropathologica, 112(4):389–404, 2006. 16906426 10.1007/s00401-006-0127-z PMC3906709

[7] Braak H. and Braak E.. Neuropathological stageing of alzheimer-related changes. Acta neuropathologica, 82(4):239–259, 1991. 1759558 10.1007/BF00308809

[8] Brodski-Guerniero A., Paasch G.-F., Wollstadt P., Özdemir I., Lizier J. T., and Wibral M.. Information-theoretic evidence for predictive coding in the face-processing system. Journal of Neuroscience, 37(34):8273–8283, 2017. 28751458 10.1523/JNEUROSCI.0614-17.2017 PMC6596791

[9] Cantarero-Prieto D., Leon P. L., Blazquez-Fernandez C., Juan P. S., and Cobo C. S.. The economic cost of dementia: a systematic review. Dementia, 19(8):2637–2657, 2020. 30909718 10.1177/1471301219837776

[10] Ciric R., Thompson W. H., Lorenz R., Goncalves M., MacNicol E., Markiewicz C. J., Halchenko Y. O., Ghosh S. S., Gorgolewski K. J., Poldrack R. A., and Esteban O.. TemplateFlow: FAIR-sharing of multi-scale, multi-species brain models. Nature Methods, 19:1568–1571, 2022. 36456786 10.1038/s41592-022-01681-2 PMC9718663

[11] Cox R. W. and Hyde J. S.. Software tools for analysis and visualization of fmri data. NMR in Biomedicine, 10(4–5):171–178, 1997. 9430344 10.1002/(sici)1099-1492(199706/08)10:4/5<171::aid-nbm453>3.0.co;2-l

[12] Dale A. M., Fischl B., and Sereno M. I.. Cortical surface-based analysis: I. segmentation and surface reconstruction. NeuroImage, 9(2):179–194, 1999. 9931268 10.1006/nimg.1998.0395

[13] Davey B. A. and Priestley H. A.. Introduction to lattices and order. Cambridge university press, 2002.

[14] Down K. J. and Mediano P. A.. Algebraic representations of entropy and fixed-sign information quantities. Entropy, 27(2):151, 2025. 40003148 10.3390/e27020151 PMC11853748

[15] Down K. J. and Mediano P. A.. A logarithmic decomposition and a signed measure space for entropy. IEEE Transactions on Information Theory, 72(1):17–41, 2025.

[16] Esteban O., Blair R., Markiewicz C. J., Berleant S. L., Moodie C., Ma F., Isik A. I., Erramuzpe A., Kent M., James D. andGoncalves E. DuPre, Sitek K. R., Gomez D. E. P., Lurie D. J., Ye Z., Poldrack R. A., and Gorgolewski K. J.. fmriprep. Software, 2018.

[17] Esteban O., Markiewicz C., Blair R. W., Moodie C., Isik A. I., Erramuzpe Aliaga A., Kent J., Goncalves M., DuPre E., Snyder M., Oya H., Ghosh S., Wright J., Durnez J., Poldrack R., and Gorgolewski K. J.. fMRIPrep: a robust preprocessing pipeline for functional MRI. Nature Methods, 2018. 10.1038/s41592-018-0235-4 PMC6319393 30532080

[18] Finn C. and Lizier J. T.. Pointwise partial information decompositionusing the specificity and ambiguity lattices. Entropy, 20(4):297, 2018. 33265388 10.3390/e20040297 PMC7512814

[19] Fonov V., Evans A., McKinstry R., Almli C., and Collins D.. Unbiased nonlinear average age-appropriate brain templates from birth to adulthood. NeuroImage, 47, Supplement 1:S102, 2009.

[20] Friston K.. The free-energy principle: a unified brain theory? Nature reviews neuroscience, 11(2):127–138, 2010. 20068583 10.1038/nrn2787

[21] Friston K., Kilner J., and Harrison L.. A free energy principle for the brain. Journal of physiology-Paris, 100(1–3):70–87, 2006. 17097864 10.1016/j.jphysparis.2006.10.001

[22] Gelens F., Äijälä J., Roberts L., Komatsu M., Uran C., Jensen M. A., Miller K. J., Ince R. A., Garagnani M., Vinck M., Distributed representations of prediction error signals across the cortical hierarchy are synergistic. Nature Communications, 15(1):3941, 2024. 10.1038/s41467-024-48329-7 PMC11087548 38729937

[23] Gorgolewski K., Burns C. D., Madison C., Clark D., Halchenko Y. O., Waskom M. L., and Ghosh S.. Nipype: a flexible, lightweight and extensible neuroimaging data processing framework in python. Frontiers in Neuroinformatics, 5:13, 2011. 21897815 10.3389/fninf.2011.00013 PMC3159964

[24] Gorgolewski K. J., Auer T., Calhoun V. D., Craddock R. C., Das S., Duff E. P., Flandin G., Ghosh S. S., Glatard T., Halchenko Y. O., The brain imaging data structure, a format for organizing and describing outputs of neuroimaging experiments. Scientific data, 3(1):1–9, 2016. 10.1038/sdata.2016.44 PMC4978148 27326542

[25] Gorgolewski K. J. Nipype. Software, 2018.

[26] Greve D. N. and Fischl B.. Accurate and robust brain image alignment using boundary-based registration. NeuroImage, 48(1):63–72, 2009. 19573611 10.1016/j.neuroimage.2009.06.060 PMC2733527

[27] Guo L.-H., Alexopoulos P., Wagenpfeil S., Kurz A., Perneczky R., Initiative A. D. N., Brain size and the compensation of alzheimer’s disease symptoms: a longitudinal cohort study. Alzheimer’s & Dementia, 9(5):580–586, 2013. 10.1016/j.jalz.2012.10.002 23232272

[28] Huntley J. D., Fleming S. M., Mograbi D. C., Bor D., Naci L., Owen A. M., and Howard R.. Understanding alzheimer’s disease as a disorder of consciousness. Alzheimer’s & Dementia: Translational Research & Clinical Interventions, 7(1):e12203, 2021. 34877398 10.1002/trc2.12203 PMC8630359

[29] Ince R. A.. Measuring multivariate redundant information with pointwise common change in surprisal. Entropy, 19(7):318, 2017.

[30] Jack C. R. Jr, Andrews J. S., Beach T. G., Buracchio T., Dunn B., Graf A., Hansson O., Ho C., Jagust W., McDade E., Revised criteria for diagnosis and staging of alzheimer’s disease: Alzheimer’s association workgroup. Alzheimer’s & Dementia, 20(8):5143–5169, 2024. 10.1002/alz.13859 PMC11350039 38934362

[31] Jack C. R. Jr, Bennett D. A., Blennow K., Carrillo M. C., Dunn B., Haeberlein S. B., Holtzman D. M., Jagust W., Jessen F., Karlawish J., Nia-aa research framework: toward a biological definition of alzheimer’s disease. Alzheimer’s & dementia, 14(4):535–562, 2018. 10.1016/j.jalz.2018.02.018 PMC5958625 29653606

[32] Jenkinson M., Bannister P., Brady M., and Smith S.. Improved optimization for the robust and accurate linear registration and motion correction of brain images. NeuroImage, 17(2):825–841, 2002. 12377157 10.1016/s1053-8119(02)91132-8

[33] Klein A., Ghosh S. S., Bao F. S., Giard J., Häme Y., Stavsky E., Lee N., Rossa B., Reuter M., Neto E. C., and Keshavan A.. Mindboggling morphometry of human brains. PLOS Computational Biology, 13(2):e1005350, 2017. 28231282 10.1371/journal.pcbi.1005350 PMC5322885

[34] Kolchinsky A.. A novel approach to the partial information decomposition. Entropy, 24(3):403, 2022. 35327914 10.3390/e24030403 PMC8947370

[35] Lanczos C.. Evaluation of noisy data. Journal of the Society for Industrial and Applied Mathematics Series B Numerical Analysis, 1(1):76–85, 1964.

[36] Larivière S., Paquola C., Park B.-y., Royer J., Wang Y., Benkarim O., Vos de Wael R., Valk S. L., Thomopoulos S. I., Kirschner M., The enigma toolbox: multiscale neural contextualization of multisite neuroimaging datasets. Nature Methods, 18(7):698–700, 2021. 34194050 10.1038/s41592-021-01186-4 PMC8983056

[37] Liardi A., Rosas F. E., Carhart-Harris R. L., Blackburne G., Bor D., and Mediano P. A.. Null models for comparing information decomposition across complex systems. arXiv preprint arXiv:2410.11583, 2024. 10.1371/journal.pcbi.1013629 PMC12614810 41191715

[38] Luppi A. I., Mediano P. A., Rosas F. E., Allanson J., Pickard J., Carhart-Harris R. L., Williams G. B., Craig M. M., Finoia P., Owen A. M., A synergistic workspace for human consciousness revealed by integrated information decomposition. Elife, 12:RP88173, 2024. 39022924 10.7554/eLife.88173 PMC11257694

[39] Luppi A. I., Mediano P. A., Rosas F. E., Holland N., Fryer T. D., O’Brien J. T., Rowe J. B., Menon D. K., Bor D., and Stamatakis E. A.. A synergistic core for human brain evolution and cognition. Nature Neuroscience, 25(6):771–782, 2022. 35618951 10.1038/s41593-022-01070-0 PMC7614771

[40] Luppi A. I., Rosas F. E., Mediano P. A., Menon D. K., and Stamatakis E. A.. Information decomposition and the informational architecture of the brain. Trends in Cognitive Sciences, 28(4):352–368, 2024. 38199949 10.1016/j.tics.2023.11.005

[41] Mattap S. M., Mohan D., McGrattan A. M., Allotey P., Stephan B. C., Reidpath D. D., Siervo M., Robinson L., and Chaiyakunapruk N.. The economic burden of dementia in low-and middle-income countries (lmics): a systematic review. BMJ global health, 7(4), 2022. 10.1136/bmjgh-2021-007409 PMC8981345 35379735

[42] Mazziotta J. C., Toga A. W., Evans A., Fox P., and Lancaster J.. A Probabilistic Atlas of the Human Brain: Theory and Rationale for Its Development: The International Consortium for Brain Mapping (ICBM). NeuroImage, 2(2, Part A):89–101, 1995. 9343592 10.1006/nimg.1995.1012

[43] Mediano P. A., Ikkala A., Kievit R. A., Jagannathan S. R., Varley T. F., Stamatakis E. A., Bekinschtein T. A., and Bor D.. Fluctuations in neural complexity during wakefulness relate to conscious level and cognition. BioRxiv, pages 2021–09, 2021.

[44] Mediano P. A., Rosas F. E., Luppi A. I., Carhart-Harris R. L., Bor D., Seth A. K., and Barrett A. B.. Toward a unified taxonomy of information dynamics via integrated information decomposition. Proceedings of the National Academy of Sciences, 122(39):e2423297122, 2025. 10.1073/pnas.2423297122 PMC12501198 40982679

[45] Moguilner S., García A. M., Mikulan E., Hesse E., García-Cordero I., Melloni M., Cervetto S., Serrano C., Herrera E., Reyes P., Weighted symbolic dependence metric (wsdm) for fmri resting-state connectivity: A multicentric validation for frontotemporal dementia. Scientific reports, 8(1):11181, 2018. 30046142 10.1038/s41598-018-29538-9 PMC6060104

[46] Motlaghian S. M., Belger A., Bustillo J. R., Ford J. M., Iraji A., Lim K., Mathalon D. H., Mueller B. A., O’Leary D., Pearlson G., Nonlinear functional network connectivity in resting functional magnetic resonance imaging data. Human brain mapping, 43(15):4556–4566, 2022. 35762454 10.1002/hbm.25972 PMC9491296

[47] Nasreddine Z. S., Phillips N. A., Bédirian V., Charbonneau S., Whitehead V., Collin I., Cummings J. L., and Chertkow H.. The montreal cognitive assessment, moca: a brief screening tool for mild cognitive impairment. Journal of the American Geriatrics Society, 53(4):695–699, 2005. 15817019 10.1111/j.1532-5415.2005.53221.x

[48] National Institute on Aging. Alzheimer’s disease: What it is and how it affects the brain and body. https://www.nia.nih.gov/health/alzheimers-and-dementia/what-alzheimers-disease, 2025. Accessed: 2026-02-11.

[49] Patriat R., Reynolds R. C., and Birn R. M.. An improved model of motion-related signal changes in fMRI. NeuroImage, 144, Part A:74–82, Jan. 2017. 27570108 10.1016/j.neuroimage.2016.08.051 PMC5533292

[50] Power J. D., Mitra A., Laumann T. O., Snyder A. Z., Schlaggar B. L., and Petersen S. E.. Methods to detect, characterize, and remove motion artifact in resting state fmri. NeuroImage, 84(Supplement C):320–341, 2014. 23994314 10.1016/j.neuroimage.2013.08.048 PMC3849338

[51] Rebbin D., Down K. J., Varley T. F., Ince R., and Canales-Johnson A.. Translating measures onto mechanisms: The cognitive relevance of higher-order information. arXiv preprint arXiv:2512.02671, 2025.

[52] Reuter M., Rosas H. D., and Fischl B.. Highly accurate inverse consistent registration: A robust approach. NeuroImage, 53(4):1181–1196, 2010. 20637289 10.1016/j.neuroimage.2010.07.020 PMC2946852

[53] Roberts L., Äijälä J., Burger F., Uran C., Jensen M. A., Miller K. J., Ince R. A., Vinck M., Hermes D., and Canales-Johnson A.. Broadband synergy versus oscillatory redundancy in the visual cortex. bioRxiv, pages 2025–02, 2025. 10.1038/s41467-026-72444-2 PMC13294381 42049731

[54] Rosas F. E., Mediano P. A., Luppi A. I., Varley T. F., Lizier J. T., Stramaglia S., Jensen H. J., and Marinazzo D.. Disentangling high-order mechanisms and high-order behaviours in complex systems. Nature Physics, 18(5):476–477, 2022.

[55] Rosas F. E., Mediano P. A., Rassouli B., and Barrett A. B.. An operational information decomposition via synergistic disclosure. Journal of Physics A: Mathematical and Theoretical, 53(48):485001, 2020.

[56] Routier A., Burgos N., Díaz M., Bacci M., Bottani S., El-Rifai O., Fontanella S., Gori P., Guillon J., Guyot A., Clinica: An open-source software platform for reproducible clinical neuroscience studies. Frontiers in Neuroinformatics, 15:689675, 2021. 34483871 10.3389/fninf.2021.689675 PMC8415107

[57] Samper-González J., Burgos N., Bottani S., Fontanella S., Lu P., Marcoux A., Routier A., Guillon J., Bacci M., Wen J., Reproducible evaluation of classification methods in alzheimer’s disease: Framework and application to mri and pet data. NeuroImage, 183:504–521, 2018. 30130647 10.1016/j.neuroimage.2018.08.042

[58] Satterthwaite T. D., Elliott M. A., Gerraty R. T., Ruparel K., Loughead J., Calkins M. E., Eickhoff S. B., Hakonarson H., Gur R. C., Gur R. E., and Wolf D. H.. An improved framework for confound regression and filtering for control of motion artifact in the preprocessing of resting-state functional connectivity data. NeuroImage, 64(1):240–256, 2013. 22926292 10.1016/j.neuroimage.2012.08.052 PMC3811142

[59] Schaefer A., Kong R., Gordon E. M., Laumann T. O., Zuo X.-N., Holmes A. J., Eickhoff S. B., and Yeo B. T.. Local-global parcellation of the human cerebral cortex from intrinsic functional connectivity mri. Cerebral cortex, 28(9):3095–3114, 2018. 28981612 10.1093/cercor/bhx179 PMC6095216

[60] Shannon C. E.. A mathematical theory of communication. The Bell system technical journal, 27(3):379–423, 1948.

[61] Tian Y., Margulies D. S., Breakspear M., and Zalesky A.. Topographic organization of the human subcortex unveiled with functional connectivity gradients. Nature neuroscience, 23(11):1421–1432, 2020. 32989295 10.1038/s41593-020-00711-6

[62] Timme N. M. and Lapish C.. A tutorial for information theory in neuroscience. eneuro, 5(3), 2018. 10.1523/ENEURO.0052-18.2018 PMC6131830 30211307

[63] Tustison N. J., Avants B. B., Cook P. A., Zheng Y., Egan A., Yushkevich P. A., and Gee J. C.. N4itk: Improved n3 bias correction. IEEE Transactions on Medical Imaging, 29(6):1310–1320, 2010. 20378467 10.1109/TMI.2010.2046908 PMC3071855

[64] Van Essen D. C., Smith S. M., Barch D. M., Behrens T. E., Yacoub E., Ugurbil K., Consortium W.-M. H., The wu-minn human connectome project: an overview. Neuroimage, 80:62–79, 2013. 23684880 10.1016/j.neuroimage.2013.05.041 PMC3724347

[65] Van Loenhoud A. C., Groot C., Bocancea D. I., Barkhof F., Teunissen C., Scheltens P., Van De Flier W. M., and Ossenkoppele R.. Association of education and intracranial volume with cognitive trajectories and mortality rates across the alzheimer disease continuum. Neurology, 98(16):e1679–e1691, 2022. 35314498 10.1212/WNL.0000000000200116 PMC9052567

[66] Van Loenhoud A. C., Groot C., Vogel J. W., Van Der Flier W. M., and Ossenkoppele R.. Is intracranial volume a suitable proxy for brain reserve? Alzheimer’s research & therapy, 10(1):91, 2018. 10.1186/s13195-018-0408-5 PMC6134772 30205838

[67] Williams P. L. and Beer R. D.. Nonnegative decomposition of multivariate information. arXiv preprint arXiv:1004.2515, 2010.

[68] Wolf H., Julin P., Gertz H.-J., Winblad B., and Wahlund L.-O.. Intracranial volume in mild cognitive impairment, alzheimer’s disease and vascular dementia: evidence for brain reserve? International journal of geriatric psychiatry, 19(10):995–1007, 2004. 15449362 10.1002/gps.1205

[69] World Health Organization. Dementia. https://www.who.int/news-room/fact-sheets/detail/dementia, 2023. Accessed 11 February 2026.

[70] Xia M., Wang J., and He Y.. Brainnet viewer: a network visualization tool for human brain connectomics. PloS one, 8(7):e68910, 2013. 23861951 10.1371/journal.pone.0068910 PMC3701683

[71] Yeo B. T., Krienen F. M., Sepulcre J., Sabuncu M. R., Lashkari D., Hollinshead M., Roffman J. L., Smoller J. W., Zöllei L., Polimeni J. R., The organization of the human cerebral cortex estimated by intrinsic functional connectivity. Journal of neurophysiology, 2011. 10.1152/jn.00338.2011 PMC3174820 21653723

[72] Zhang X., Han C., Xia J., Deng L., and Dong J.. Dynamic synergy network analysis reveals stage-specific regional dysfunction in alzheimer’s disease. Brain Sciences, 15(6):636, 2025. 40563806 10.3390/brainsci15060636 PMC12190503

[73] Zhang Y., Brady M., and Smith S.. Segmentation of brain MR images through a hidden markov random field model and the expectation-maximization algorithm. IEEE Transactions on Medical Imaging, 20(1):45–57, 2001. 11293691 10.1109/42.906424

